# Coevaporated Formamidinium Tin Triiodide with Suppressed p‑Type Self-Doping

**DOI:** 10.1021/acsenergylett.5c03400

**Published:** 2025-12-12

**Authors:** Junhyoung Park, Andrea Olivati, Mirko Prato, Min Kim, Annamaria Petrozza

**Affiliations:** † Center for Nano Science and Technology@Polimi, 121451 Istituto Italiano di Tecnologia, via Rubattino 81, Milano 20134, Italy; ‡ Materials Characterization Facility, 121451 Istituto Italiano di Tecnologia, Via Morego, Genova 16163 Italy; § Department of Chemical Engineering, 35010 University of Seoul, 163, Seoulsiripdae-ro, Dongdaemun-gu, Seoul 02504, South Korea

## Abstract

Coevaporation of formamidinium tin triiodide (FASnI_3_) precursors, without any additives or reducing agents, leads to the growth of a highly crystalline thin films which show a bandgap around 1.31 eV, closely matching the theoretical value predicted from the ideal single crystal structure of FASnI_3_. The polycrystalline thin film presents a lower tendency toward Sn^2+^ to Sn^4+^ oxidation and highly reduced tendency toward self-doping, demonstrating, overall, an improved resistance to defects formation. These findings suggest solvent-free coevaporation processes as a promising route for high quality Sn-based perovskite polycrystalline thin films.

## Graphical abstract

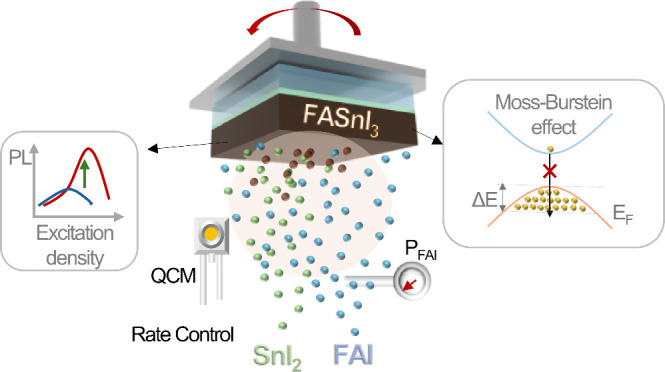

Metal halide perovskites are revolutionizing optoelectronic applications, including solar cells, LED, and photodetectors, due to their exceptional optical properties. Tin halide perovskites (THPs) are regarded as promising candidates for applications such as solar cells, near-infrared LEDs, and photodetectors due to their narrower bandgaps compared to lead halide perovskites (LHPs). However, chemically active lone pair electrons and low Sn-vacancy (V_Sn_) formation energy promote Sn^2+^ to Sn^4+^ oxidation. This causes self-doping of THPs that exhibit p-type semiconductor characteristics. While, *per se,* it is a feature which may be exploited in different (opto)­electronic and photonic applications, the self-doping is currently not under control, which makes THPs semiconductors not a reliable class of materials. So far, solution-processed THPs thin films development has been primarily conducted via additive engineering with the attempt of controlling defects formation. With all efforts, THPs still suffer from chemical instability due to inevitable solvent-induced defects and remnant molecules. Consequently, solvent-free evaporation techniques, also offering precise stoichiometry control, large-area uniformity, and processing in O_2_/moisture-free environment, are especially advantageous for THPs fabrication, enabling constraint of solvent related issues.

However, only a few attempts have been shown in this direction, without a detailed characterization of the thin film properties.^1^ Specifically, coevaporated methylammonium tin triiodide (MASnI_3_) has shown poor morphology and optical characteristics than the spin-coated one.^2,3^ Although it is not clear, this might be due to the diffusive nature of MAI evaporation and the inherent instability of MA^+^. In contrast, formamidinium (FA^+^)-based perovskites generally have exhibited higher thermal stability and performance compared to MA^+^-based ones, attributable to lower volatility and reduced fragmentation of FA^+^. Despite this, the successful coevaporation of FASnI_3_ and investigation on its properties remain unreported.

Here, we report the first highly crystalline, additive-free, coevaporated pure FASnI_3_ film. We compare stoichiometric coevaporated and spin-coated FASnI_3_ (with SnF_2_) films to highlight their distinct material and optical properties. As shown in Figure 1a and Figure S1, coevaporated films exhibited a pinhole free flat surface with compact morphology compared to spin-coated ones. X-ray diffraction (XRD) patterns (Figure 1b) show that coevaporated FASnI_3_ peaks closely match simulated patterns for ideal cubic (*Pm*3̅*m*), unlike significant deviations in spin-coated peaks.^4,5^ (Figure S2). The shift of XRD peaks toward higher angles and reduced full width at half-maximum (FWHM) in coevaporated films imply they experience reduced nonuniform lattice strain, likely due to more homogeneous crystallization on the substrate.^6^ In contrast, the spin-coated film may experience greater structural disorder and tensile strain.^7^

**Figure 1:**
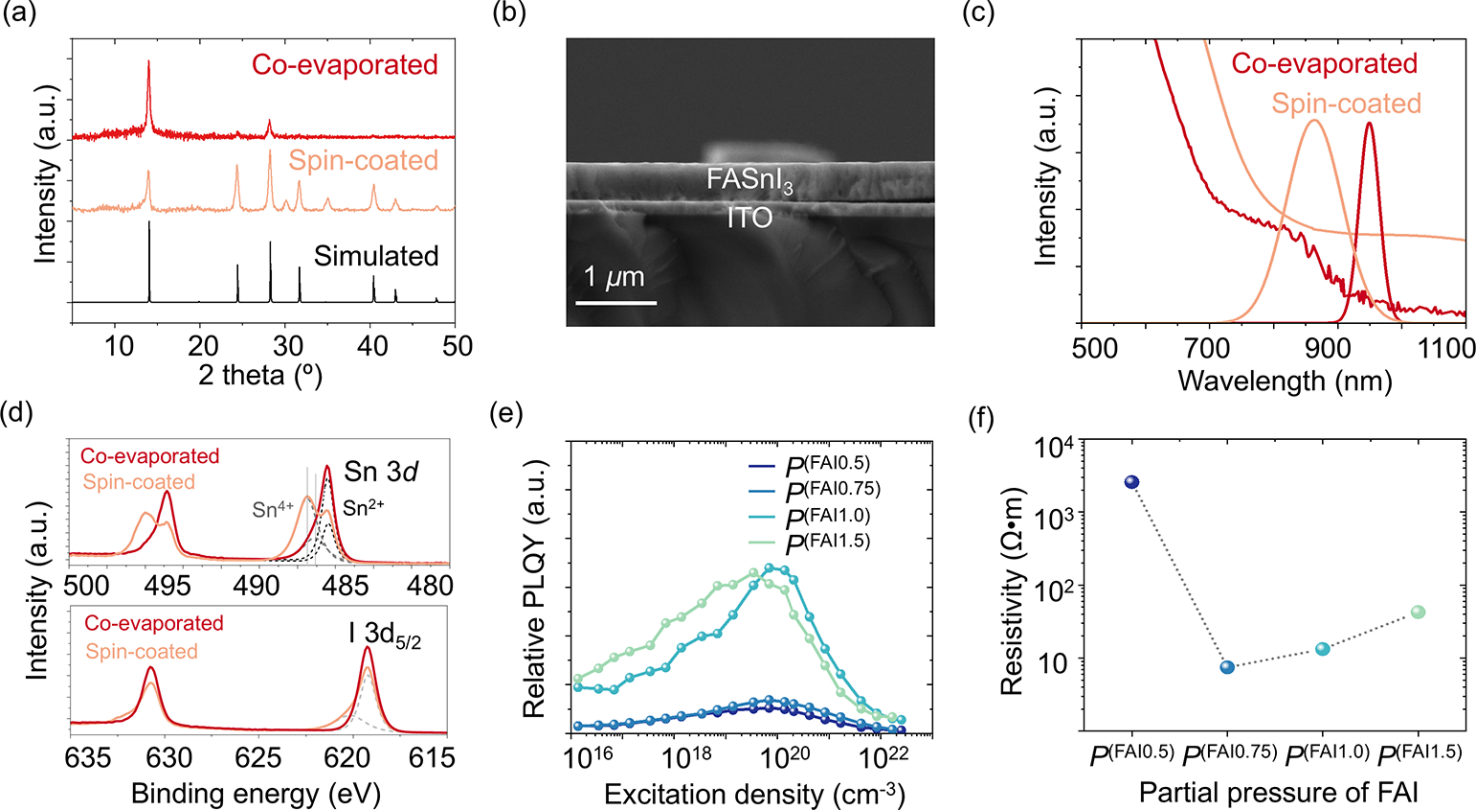
(a) X-ray diffraction (XRD) patterns comparing three cases: an ideal cubic structure calculated computationally, an experimentally, coevaporated FASnI_3_ film and spin-coated (with SnF_2_). (b) Cross-sectional SEM image of the coevaporated FASnI_3_ film (∼400 nm thickness), demonstrating a highly uniform and smooth surface. (c) Optical characterization through absorption and photoluminescence spectra of coevaporated and spin-coated FASnI_3_ (with SnF_2_) films. (d) X-ray photoelectron spectroscopy (XPS) spectra showing the binding energies of Sn 3d_5/2_ and I 3d_5/2_ states in coevaporated FASnI_3_. (e) Excitation density dependent Photoluminescence Quantum Yield (PLQY) of coevaporated FASnI_3_ films fabricated under conditions *P* ^(FAI0.5)^, *P* ^(FAI0.75)^, *P* ^(FAI1.0)^, and *P* ^(FAI1.5)^. (f) Resistivity of coevaporated FASnI_3_ films corresponding to conditions *P* ^(FAI0.5)^, *P* ^(FAI0.75)^, *P* ^(FAI1.0)^, and *P* ^(FAI1.5)^.

Optical characterizations further support this structural distinction. The spin-coated film exhibits a blue-shifted absorption onset and high background (∼850 nm, Figure 1c), suggesting high doping levels and broader distribution of sub-bandgap states.^8^ In contrast, the coevaporated film shows a sharper absorption edge, implying well-defined density of states near the edge. Consistently, the PL spectrum of coevaporated film shows minimal blue-shift with narrow FWHM (37.2 nm), suggesting a negligible Moss-Burstein effect, indicative of reduced electronic doping, while spin-coated one show broad and blue-shifted PL spectrum. A Tauc plot analysis further reveals the coevaporated film has a narrower optical bandgap (∼1.31 eV) compared to previously reported state-of-the-art spin-coated films (∼1.40 eV, Figure S3).^9^ This result is also consistent with DFT calculations (HSE06-D3) for FASnI_3_ bandgap.^10^ From a combined understanding, we propose that the tensile strain in spin-coated films reduces the overlap between Sn 5s and I 5p orbitals, thereby increasing the energy gap between bonding and antibonding states.

XPS analysis reveals clear differences in surface chemical characteristics between the films, as shown in Figure 1d. The coevaporated film predominantly showed Sn^2+^ signals (Sn 3d_5/2_ BE: 486.4 ± 0.2 eV), while spin-coated FASnI_3_ exhibited significant Sn^4+^ states (as revealed by the presence of a Sn 3d_5/2_ peak at approx. 487.5 eV), consistent with prior reports (Figure 1c). Instead, a minor component, which was not clearly resolved, appeared at 487.1 eV in the coevaporated film.^11^ For I 3d_5/2_, coevaporated films displayed a single peak (centered at approximately 619.2 eV), whereas spin-coated films showed two peaks (619.2 and 620.2 eV), suggesting rapid surface degradation even under strictly controlled conditions (O_2_/H_2_O < 0.5 ppm). This suppressed Sn^4+^ formation consistently indicates minimal Sn-vacancy formation in coevaporated films, reducing charge-compensating defects and background holes. UPS results (Figure S4) corroborate this, showing a midgap Fermi level, confirming close to intrinsic semiconductor characteristics of coevaporated films.

Further, to show p-doping controllability of coevaporation, we investigated material/optical property dependence on FAI partial pressure (*P* ^FAI^). Recognizing that precursor interactions strongly affect sticking coefficients,^12,13^ four distinct deposition rate ratios (FAI/SnI_2_) were nominally defined—*P* ^(FAI0.5)^, *P* ^(FAI0.75)^, *P* ^(FAI1.0)^, and *P* ^(FAI1.5)^—reflecting *P* ^FAI^ (Figure S5). *P* ^FAI^ markedly influences the microstructural, crystallographic, optical, and electrical properties. While all conditions produced highly absorptive films, substantial differences in microstructure and crystallinity emerged (Figures S6 and S7a). Conditions *P* ^(FAI0.75)^ and *P* ^(FAI1.0)^ yield highly crystalline and uniformly structured films. In contrast, *P* ^(FAI0.5)^ and *P* ^(FAI1.5)^ led to amorphous phases without a long-range order.

Optical analysis further elucidates the structural quality dependence on *P* ^FAI^ (Figure S7b). Films deposited under *P* ^(FAI1.0)^ exhibit the steepest Urbach tail, indicative of superior crystallinity, whereas flatter Urbach tails observed for *P* ^(FAI0.5)^ and *P* ^(FAI1.5)^ signified energetic disorder, consistent with structural analyses. Correspondingly, PL spectra (Figure S7c) indicate emission peaks at longer wavelengths (∼950 nm, 1.31 eV) specifically for *P* ^(FAI1.0)^, affirming a minimal Moss-Burstein effect from a reduced self-doping. Excitation-dependent PLQY measurements elucidate defects and doping effects. *P* ^(FAI0.5)^ and *P* ^(FAI0.75)^ showed lower relative PLQY due to dominant nonradiative recombination (Figure 1e). *P* ^(FAI1.0)^ exhibited the highest relative PLQY. Interestingly, all samples show a power dependence of the PLQY, though with different slopes, until Auger-like process kick-in. This is well in agreement with low electronic doping densities. Accordingly, resistivity measurements (Figure 1f) also explain the effect of doping. Films deposited at *P* ^(FAI0.5)^, *P* ^(FAI0.75)^, *P* ^(FAI1.0)^, and *P* ^(FAI1.5)^ exhibited resistivities of approximately 2.57 kΩ·m, 7.4 Ω·m, 13.3 Ω·m, and 42.5 Ω·m, respectively, substantially higher than those typically observed in spin-coated FASnI_3_ (without SnF_2_: 10^–2^-10^–4^ Ω·m^11^ and with 10% of SnF_2_: 1.3 × 10^–2^ Ω·m^14,15^ ). Higher resistivity indicates a decreased hole concentration from self-doping.

This systemic research demonstrates coevaporation—when performed without additives—can produce an FASnI_3_ film with restrained p-doping. Coevaporated films exhibit ideal optical and structural properties, closely matching computational predictions, highlighting the significant influence of coevaporation on THP quality. It deepens understanding of FASnI_3_ coevaporation, establishing it as a promising route for high-quality THP and offering a fundamental framework for extending into optoelectronic applications.

## Supplementary Material



## Notes

General synthetic method (Method section); cross-section/surface images (Figure S1); XRD patterns (Figure S2); Tauc plots (Figure S3); UPS data and estimated CBM/VBM (Figure S4); total pressure of vacuum chamber while evaporation (Figure S5); surface image of FASnI_3_ coevaporated in different partial pressure conditions (Figure S6); XRD, Tauc plot, and PL spectra of FASnI_3_ coevaporated in different partial pressure conditions (Figure S7) (PDF)

## Abbreviations

THP: Tin halide perovskite
LHP: Lead halide perovskite
MASnI_3_: Methylammonium tin triiodide
FASnI_3_: Formamidinium tin triiodide
FAI: Formamidinium iodide
MAI: Methylammonium iodide
PLQY: Photoluminescence quantum yield
