# Supplementary material for: Coevaporated Formamidinium Tin Triiodide with Suppressed p‑Type Self-Doping
Source: ACS Energy Lett. 2025 Dec 12;11(1):374–7. doi: 10.1021/acsenergylett.5c03400 (PMC12797832; doi:10.1021/acsenergylett.5c03400)
Supplement: Supplementary file 1 [file nz5c03400_si_001.pdf]

Supporting Information

Co-evaporated Formamidinium tin triiodide with suppressed p-type self-doping

Junhyoung Park[1], Andrea Olivati[1], Mirko Prato[2], Min Kim[3], Annamaria Petrozza[1]

[1] Center for Nano Science and Technology@Polimi, Istituto Italiano di Tecnologia, via Rubattino 81, Milano 20134, Italy

[2] Materials Characterization Facility, Istituto Italiano di Tecnologia, Via Morego, Genova, 16163 Italy

[3]Department of Chemical Engineering, University of Seoul, 163, Seoulsiripdae-ro, Dongdaemun-gu, Seoul, 02504, South Korea

Methods

Chemicals All the commercial materials were used as received, SnI_2 (Anhydro beads, −10 mesh, 99.99% trace metals basis, Sigma-Aldrich), SnF_2 (99%, Sigma-Aldrich), Formamidinium iodide (>99.99%, greatcellsolar materials), *N, N*-dimethylformamide (99.99%, Sigma-Aldrich), dimethyl sulfoxide (99.50%, Sigma-Aldrich), chlorobenzene (99.8%, Sigma-Aldrich)

Sample preparation Indium tin oxide (ITO)-deposited glass substrates (4-5 Ω/sq) were precisely patterned to dimensions of 25×25 mm. The substrates underwent a rigorous cleaning procedure involving sequential ultra-sonication for 30 minutes each in detergent (Hellmanex), acetone, de-ionized water, and isopropanol. Following cleaning, the substrates were dried using a nitrogen (N_2) gun and subsequently dried in ambient air for 24 hours. To ensure the complete removal of organic contaminants prior to perovskite deposition, the substrates were subjected to UV-ozone treatment for 15 minutes. Perovskite layers were deposited via thermal co-evaporation within high-vacuum chambers maintained inside a N_2-filled glovebox, where the H_2O and O_2 levels were strictly controlled to <0.5 ppm. The vacuum chambers were evacuated to a base pressure of 5×10^{-7} mbar using turbomolecular pumps. The chambers were equipped with four temperature-controlled evaporation sources, each fitted with a ceramic crucible. These sources were directed vertically upwards (90° angle) toward the substrate holder. Four dedicated quartz crystal microbalance (QCM) sensors continuously monitored the deposition rate of each source. The perovskite precursors, formamidinium iodide (FAI) and tin (II) iodide (SnI_2), were loaded into two crucibles and thermally evaporated. Initial evaporation rates of 0.1 A°/s for both precursors were achieved by heating FAI to approximately 130 °C and SnI_2 to 150 °C. After stabilization, the temperature of the source increases to achieve target rate. $FASnI_3$ thin films were also fabricated using a one-step spin coating procedure. The 1.2 M precursor solution was prepared in a mixed solvent system of *N, N*-dimethyl formamide (DMF) and dimethyl sulfoxide (DMSO) with a volume ratio of 4:1. Tin (II) fluoride (SnF_2) was incorporated as an additive at 10 mol% relative to the SnI_2 concentration. The solution was prepared 6 hours prior to use and filtered through a 0.20 μm PTFE membrane immediately before spin coating. Films were deposited by spinning the substrate at 5000 rpm for 50 seconds. Chlorobenzene (300 μl) was introduced onto the spinning substrate 20 seconds before the end of the process. The resulting films were then annealed at 100°C for 20 minutes. To prevent degradation from environmental exposure, the thin films were immediately encapsulated inside the glovebox using a Nagase ChemteX UV-curable adhesive, which achieved curing within 5 min under UV light. This sealing procedure ensures material stability for a minimum period of one month.[1] However, all subsequent characterizations for every fabricated batch were strictly completed within five days of thin-film preparation.

Material Film Characterization The structural characterization of the samples was performed using X-ray diffraction (XRD). Patterns were measured at room temperature with a Bruker D8 Advance diffractometer utilizing Bragg-Brentano geometry and a Cu Kα1 anode (λ = 1.544060 Å), operated at 40 kV and 40 mA. Data were collected in symmetric reflection mode with a step size of 0.02 ° and an acquisition time of 1 s per step. PDXL application software, which references the ICDD PDF-2 database and VESPA, was utilized for data processing.[2] UV-Vis steady-state absorption spectra were recorded on perovskite thin films deposited on bare glass substrates using a PerkinElmer Lambda 1050 UV/VIS/NIR spectrophotometer across the wavelength range of 450–1100 nm, employing a 2 nm step size. Finally, Scanning Electron Microscopy (SEM) images were obtained from perovskite films prepared on ITO substrates using a ZEISS Gemini 360 microscope with an accelerating voltage of 6 kV. For optical characterization, the sample placed in specially designed air-tight optical holder was excited using the 515 nm second harmonic generated by a Light Conversion Pharos fundamental laser. The excitation power density was set to approximately 100 mW cm^{-2}. The resulting photoluminescence (PL) signal was then collected in a reflection configuration (perpendicular to the excitation beam), coupled into an optical fiber, and spectrally resolved by an Ocean Optics Maya Pro 2000 spectrometer.

X-ray and Ultraviolet photoelectron spectroscopy (XPS & UPS): XPS was carried out on a Kratos Axis $Ultra^{DLD}$ spectrometer. Wide scans were acquired at a pass energy of 160 eV, with an energy step of 1 eV, over a (300 x 700) μm^2 area. High-resolution spectra were acquired at a pass energy of 10 eV and energy step of 0.1 eV, over the same analysis area. All the spectra were obtained using a monochromatic Al Kα source (15 kV, 20 mA). XPS data were analyzed using CasaXPS (version 2.3.24)[3] To account for possible charging effects, the N 1s peak due to the C-N bonds in formamidinium was used as an internal reference for the binding energy scale, and its position was set to 400.6 eV, in agreement with literature reports.[4]

UPS was carried out on the same spectrometer, using a He I (21.22 eV) discharge lamp, on an area of 55 μm in diameter, at a pass energy of 10 eV and with a dwell time of 100 ms. The work function (that is, the position of the Fermi level with respect to the vacuum level) was measured from the threshold energy for the emission of secondary electrons during He I excitation. A −9.0 V bias was applied to the sample to precisely determine the low-kinetic-energy cutoff, as discussed in ref [5].The position of the cutoff was then estimated with CasaXPS software, using the "Edge Up" background function for the energy interval around the cutoff. Then, the position of the VBM versus the vacuum level was estimated by measuring its distance from the Fermi level, focusing on the high-kinetic energy (i.e., low-binding energy) cutoff region and using the "Edge Down" background function in CasaXPS software.

(1) Park, J.; Byeon, J.; Jang, J.; Ko, M.; Ahn, N.; Choi, M.; Song, H.-J. Electrically Reliable Perovskite Photovoltaic Cells Against Instantaneous Kilovolt Stress. *Advanced Energy Materials* **2023**, *13* (3), 2203012. DOI: https://doi.org/10.1002/aenm.202203012.

(2) Park, J.; Kim, S.; Chu, Y. H.; Lee, J.; Son, D.-y.; Choi, M.; Lee, Y. S. De-Intercalation of Iodoplumbate(DMSO)x Complex for Uniaxially Oriented Halide Perovskite Thin-Film Solar Cells. *Advanced Energy Materials* **2024**, *14* (39), 2400620. DOI: https://doi.org/10.1002/aenm.202400620.

(3) Fairley, N.; Fernandez, V.; Richard-Plouet, M.; Guillot-Deudon, C.; Walton, J.; Smith, E.; Flahaut, D.; Greiner, M.; Biesinger, M.; Tougaard, S.; et al. Systematic and collaborative approach to problem solving using X-ray photoelectron spectroscopy. *Applied Surface Science Advances* **2021**, *5*, 100112. DOI: https://doi.org/10.1016/j.apsadv.2021.100112.

(4) Huerta Hernandez, L.; Haque, M. A.; Sharma, A.; Lanzetta, L.; Bertrandie, J.; Yazmaciyan, A.; Troughton, J.; Baran, D. The role of A-site composition in the photostability of tin–lead perovskite solar cells. *Sustainable Energy & Fuels* **2022**, *6* (20), 4605–4613, 10.1039/D2SE00663D. DOI: 10.1039/D2SE00663D.

(5) Helander, M. G.; Greiner, M. T.; Wang, Z. B.; Lu, Z. H. Pitfalls in measuring work function using photoelectron spectroscopy. *Applied Surface Science* **2010**, *256* (8), 2602–2605. DOI: https://doi.org/10.1016/j.apsusc.2009.11.002.

(6) Siebentritt, S.; Rau, U.; Gharabeiki, S.; Weiss, T. P.; Prot, A.; Wang, T.; Adeleye, D.; Drahem, M.; Singh, A. Photoluminescence assessment of materials for solar cell absorbers. *Faraday Discussions* **2022**, *239* (0), 112–129, 10.1039/D2FD00057A. DOI: 10.1039/D2FD00057A.

(7) Poli, I.; Ambrosio, F.; Treglia, A.; Berger, F. J.; Prato, M.; Albaqami, M. D.; De Angelis, F.; Petrozza, A. Photoluminescence Intensity Enhancement in Tin Halide Perovskites. *Advanced Science* **2022**, *9* (32), 2202795. DOI: https://doi.org/10.1002/advs.202202795.

(8) Poli, I.; Kim, G.-W.; Wong, E. L.; Treglia, A.; Folpini, G.; Petrozza, A. High External Photoluminescence Quantum Yield in Tin Halide Perovskite Thin Films. *ACS Energy Letters* **2021**, *6* (2), 609–611. DOI: 10.1021/acsenergylett.0c02612.

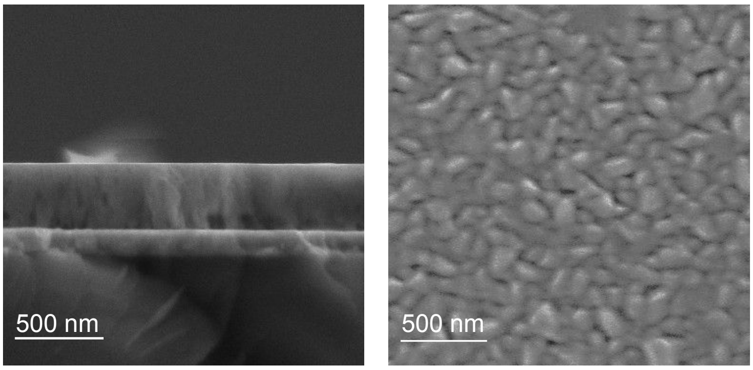

Figure S1. Enlarged Cross-section image and surface microstructure of co-evaporated thin films with thickness of ~400 nm. Microstructure shows smooth and compact grain size of around 200 nm.

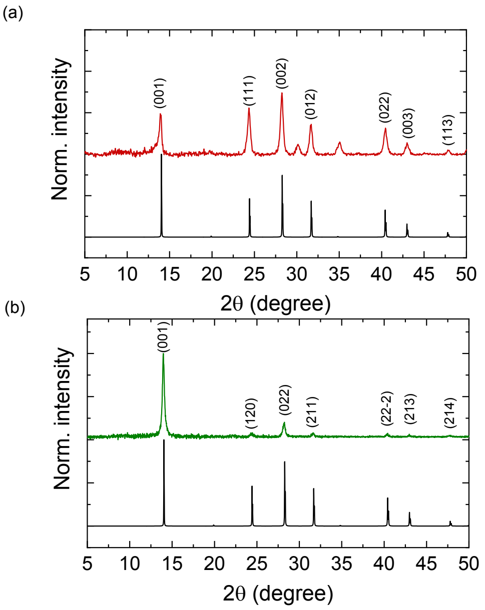

Spin-coated			Ortho (Simulated) *Amm2*			Cubic (Simulated) Pm-3m		
2 θ	d (Å)		2 θ	d (Å)	*Δ*		c	d (Å)	*Δ*
**13.88**	6.35	(100)	13.98	6.33	0.1	(001)	**14.03**	6.31	-0.13
24.51	3.62	(120)	24.33	3.66	0.03	(111)	24.42	3.64	-0.06
28.35	3.13	(022)	28.18	3.1	0.05	(002)	28.28	3.15	-0.05
31.79	2.79	(211)	31.59	2.83	0.07	(012)	31.7	2.82	-0.04

Co-evaporated			Ortho (Simulated) *Amm2*			Cubic (Simulated) *Pm-3m*		
2 θ	*d* (Å)		*2 θ*	*d* (Å)	*Δ*		*2 θ*	*d* (Å)	*Δ*
**13.97**	6.32	(100)	13.98	6.33	**-0.01**	(001)	**14.03**	6.31	-0.06
24.38	3.62	(120)	24.33	3.66	**0.05**	(111)	24.42	3.64	-0.04
28.19	3.13	(022)	28.18	3.17	**0.01**	(002)	28.28	3.15	-0.09
31.62	2.79	(211)	31.59	2.83	**0.03**	(012)	31.7	2.82	-0.08

Figure S2. (a) X-ray diffraction patterns of spin-coated pure $FASnI_3$. The peak position gap between computationally calculated ideal orthorhombic (*Amm2*) and cubic ($Pm\bar{3}m$) are huge. (b) X-ray diffraction patterns of co-evaporated $FASnI_3$ which are close to ideal structure calculated from ideal orthorhombic (*Amm2*) and cubic ($Pm\bar{3}m$).

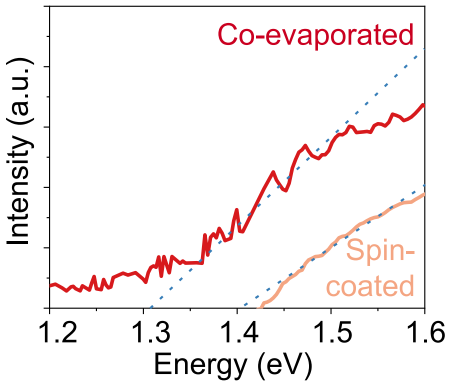

Figure S3. Bandgaps estimated from absorption curve using Tauc method of Co-evaporated $FASnI_3$ films and Spin-coated (with SnF_2).

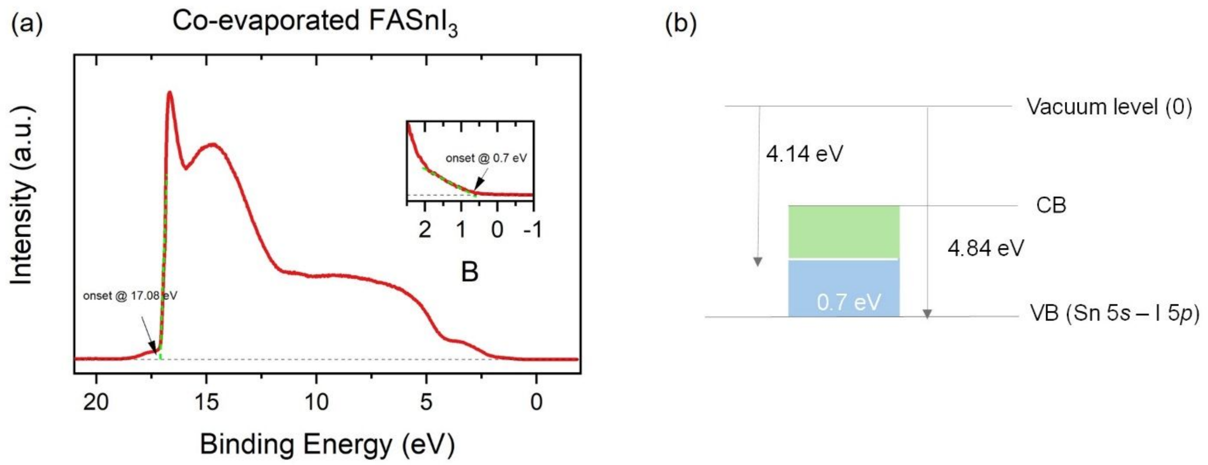

Figure S4. (a) Ultra-violet photoelectron spectroscopy measured on co-evaporated $FASnI_3$. (b) Estimated conduction band minima and Valence band maxima energy estimated from UPS measurements. The correct fittings of the spectra were conducted using Casa XPS software and built-in algorism for fitting.

	**Total pressure (mTorr)**
$P^{(FAI1.5)}$	4~6*10^{-6}
$P^{(FAI1.0)}$	1.6 ~2.5*10^{-6}
$P^{(FAI0.75)}$	1.2 ~1.4 *10^{-6}
$P^{(FAI0.5)}$	0.9 ~1.2 *10^{-6}

Figure S5. Total pressure was tracked using PKR 251 absolute pressure sensor (Pirani/Penning) horizontally installed approximately same height with the substrate in the evaporator. The pressure of the vacuum was Pfeiffer Display control unit 110.

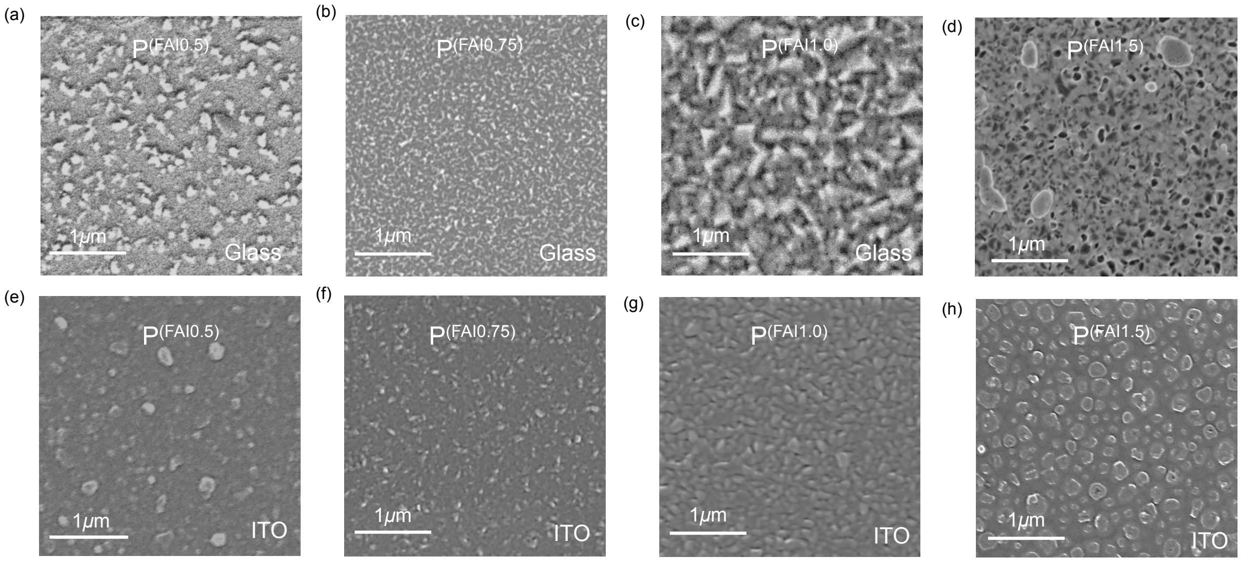

Figure S6. (a) $FASnI_3$ film fabricated on glass under $P^{(\mathrm{FAI0.5})}$ condition. (b) under $P^{(\mathrm{FAI0.75})}$ condition. (c) $P^{(\mathrm{FAI1.0})}$ condition (d) $P^{(\mathrm{FAI1.5})}$ condition. (e) $FASnI_3$ film fabricated on ITO glass under $P^{(\mathrm{FAI0.5})}$ condition. (f) $FASnI_3$ under $P^{(\mathrm{FAI0.75})}$ condition. (g) under $P^{(\mathrm{FAI1.0})}$ condition. (h) under $P^{(\mathrm{FAI1.5})}$ condition.

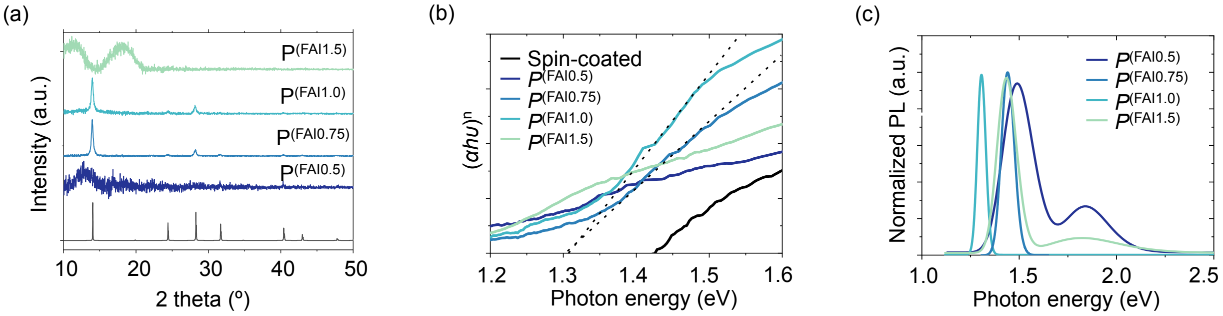

Figure S7. (a) X-ray diffraction patterns were obtained from co-evaporated $FASnI_3$ films fabricated within different partial pressure of FAI. These films were prepared under four distinct partial pressures of formamidinium iodide (FAI), denoted in increasing order of partial pressure as $P^{(\mathrm{FAI0.5})}$, $P^{(\mathrm{FAI0.75})}$, $P^{(\mathrm{FAI1.0})}$, and $P^{(\mathrm{FAI1.5})}$ (b) Tauc plot analysis of co-evaporated $FASnI_3$ films prepared at four distinct partial pressures of Formamidinium iodide (FAI), denoted as $P^{(\mathrm{FAI0.5})}$, $P^{(\mathrm{FAI0.75})}$, $P^{(\mathrm{FAI1.0})}$, and $P^{(\mathrm{FAI1.5})}$. (c) Photoluminescence spectra plotted against photon energy for co-evaporated $FASnI_3$ films fabricated under conditions $P^{(\mathrm{FAI0.5})}$, $P^{(\mathrm{FAI0.75})}$, $P^{(\mathrm{FAI1.0})}$, and $P^{(\mathrm{FAI1.5})}$.
